# Synthetic Peptides Elicit Strong Cellular Immunity in Visceral Leishmaniasis Natural Reservoir and Contribute to Long-Lasting Polyfunctional T-Cells in BALB/c Mice

**DOI:** 10.3390/vaccines7040162

**Published:** 2019-10-28

**Authors:** Rory Cristiane Fortes De Brito, Jamille Mirelle de Oliveira Cardoso, Levi Eduardo Soares Reis, Fernando Augusto Siqueira Mathias, Rodrigo Dian de Oliveira Aguiar-Soares, Andréa Teixeira-Carvalho, Bruno Mendes Roatt, Rodrigo Corrêa-Oliveira, Jeronimo Conceição Ruiz, Daniela de Melo Resende, Alexandre Barbosa Reis

**Affiliations:** 1 Laboratório de Imunopatologia, Núcleo de Pesquisas em Ciências Biológicas/NUPEB, Universidade Federal de Ouro Preto, 35400-00 Ouro Preto, Minas Gerais, Brazil; rorybrito@gmail.com (R.C.F.D.B.); ja_mirelle@yahoo.com.br (J.M.d.O.C.); levieduardo@yahoo.com.br (L.E.S.R.); fa_mathias@yahoo.com.br (F.A.S.M.); rodrigodian@gmail.com (R.D.d.O.A.-S.); bmroatt@gmail.com (B.M.R.); 2 Laboratório Multiusuário de Citometria de Fluxo, Núcleo de Pesquisas em Ciências Biológicas/NUPEB, Universidade Federal de Ouro Preto, 35400-00 Ouro Preto, Minas Gerais, Brazil; 3 Laboratório de Biomarcadores de Diagnóstico e Monitoração, Instituto René Rachou, Fundação Oswaldo Cruz, 30190-002 Belo Horizonte, Minas Gerais, Brazil; atcteixeira@gmail.com; 4 Instituto Nacional de Ciência e Tecnologia em Doenças Tropicais (INCT-DT), 40110-160 Salvador, Brazil; 5 Departamento de Ciências Biológicas, Instituto de Ciências Exatas e Biológicas, Universidade Federal de Ouro Preto, 35400-00 Ouro Preto, Minas Gerais, Brazil; 6 Grupo Imunologia Celular e Molecular, Programa de Pós-Graduação em Ciências da Saúde, Instituto René Rachou, Fiocruz Minas, 30190-002 Belo Horizonte, Minas Gerais, Brazil; rodrigo.correa@fiocruz.br; 7 Grupo Informática de Biossistemas e Genômica, Programa de Pós-Graduação em Ciências da Saúde, Instituto René Rachou, Fiocruz Minas, 30190-002 Belo Horizonte, Minas Gerais, Brazil; jeronimo.ruiz@fiocruz.br (J.C.R.); dani.melo.resende@gmail.com (D.d.M.R.); 8 Programa de Pós-Graduação em Biologia Computacional e Sistemas, Instituto Oswaldo Cruz, Fiocruz, Rio de Janeiro, 21040-360 Rio de Janeiro, Brazil; 9 Departamento de Análises Clínicas, Escola de Farmácia, Universidade Federal de Ouro Preto, 35400-00 Ouro Preto, Minas Gerais, Brazil

**Keywords:** reverse vaccinology, immunoinformatics, peptide-based vaccine, *Leishmania infantum*, polyfunctional T-cells, memory T-cells, rational design of vaccines

## Abstract

Reverse vaccinology or immunoinformatics is a computational methodology which integrates data from in silico epitope prediction, associated to other important information as, for example, the predicted subcellular location of the proteins used in the design of the context of vaccine development. This approach has the potential to search for new targets for vaccine development in the predicted proteome of pathogenic organisms. To date, there is no effective vaccine employed in vaccination campaigns against visceral leishmaniasis (VL). For the first time, herein, an in silico, in vitro, and in vivo peptide screening was performed, and immunogenic peptides were selected to constitute VL peptide-based vaccines. Firstly, the screening of in silico potential peptides using dogs naturally infected by *L. infantum* was conducted and the peptides with the best performance were selected. The mentioned peptides were used to compose Cockt-1 (cocktail 1) and Cockt-2 (cocktail 2) in combination with saponin as the adjuvant. Therefore, tests for immunogenicity, polyfunctional T-cells, and the ability to induce central and effector memory in T-lymphocytes capacity in reducing the parasite load on the spleen for Cockt-1 and Cockt-2 were performed. Among the vaccines under study, Cockt-1 showed the best results, eliciting CD4^+^ and CD8^+^ polyfunctional T-cells, with a reduction in spleen parasitism that correlates to the generation of T CD4^+^ central memory and T CD8^+^ effector memory cells. In this way, our findings corroborate the use of immunoinformatics as a tool for the development of future vaccines against VL.

## 1. Introduction

Despite several studies in vaccine development, to date there is no licensed human vaccine against leishmaniasis. Thus, there is a need for an effective and safe vaccine capable of being used in mass vaccination campaigns in the endemic areas around the world. In this view, many other strategies for identifying antigens have been suggested for the design of vaccines against leishmaniasis [1]. Among these strategies, immunoinformatics (reverse vaccinology) seems to be very promising. In this context, reverse vaccinology emerges, using various potentialities of bioinformatics in genomic or proteomic sequences, instead of pathogens, as material for the identification of new antigens, and the activity of the identified targets is confirmed later by biological experiments. In general, the objective is the identification of genes capable of expressing proteins of the pathogen with immunogenic potential, both secreted or associated with the plasmatic membrane. The information about subcellular location may be obtained from specific computational approaches. This computational methodology allows to identify proteins that are accessible to the immune system and that can mediate the development of a protective immune response [2]. Thus, there is a significant reduction in time and cost required to find new targets for vaccine development.

Additionally, recent advances in understanding the mechanisms of cellular immunity in leishmaniasis have promoted an increase in the use of peptides as immunogens for the activation of T-cells and vaccine development. The use of peptides, which was in decay until a few years ago, appears as one of the most promising approaches for the rational design of vaccines [3,4,5]. The great challenge in immunology is how to identify antigens capable of generating more long-lasting immune memory against vaccine immunizations. In this context, several approaches to assess immunological memory have been developed, and some studies using multifunctional cytometry aim to identify and evaluate central and effector memory T-lymphocytes to validate different vaccine candidates, as studies reveal that protection against *Leishmania* may be related to the generation of a specific group of memory cells, mainly the central and effector memory cells [6].

In summary, this initiative was strategically designed to propose the use of immunoinformatics to map epitopes and different approaches to the design of vaccines. Herein, we proposed the screening of peptides in the *Leishmania infantum* naturally infected canine model for the evaluation of important markers of protection. We also suggested peptide cocktail vaccines to contribute in this area of vaccine design and development against experimental visceral leishmaniasis (VL). In this sense, our study contributes to a better elucidation of protective mechanisms of peptide-based vaccines, and mechanisms related to polyfunctional and memory T-cells that lead to parasite elimination and disease control.

## 2. Materials and Methods

### 2.1. Ethical Statement

The study was carried out under the recommendation of the National Institute of Health, USA. The protocol number 2015/03 was approved by the Ethical Committee for the Use of Experimental Animals (CEUA) of the Universidade Federal de Ouro Preto, Ouro Preto, Minas Gerais, Brazil. All the experiments were made to minimize animal suffering.

### 2.2. Study Design

The study was performed as follows:

(1) Selection of linear epitopes for T-cells based on a pipeline described by Brito et al. (2017) [7]: This pipeline was used to map the whole *L. infantum* predicted proteome, comprising the selection of potential proteins that have a consensus of predicted binding epitopes to major histocompatibility complex (MHC) class I and II, B cell epitopes, and specific subcellular locations. Thus, from the results of different immunoinformatics approaches employed, we constructed a relational database integrating the data of the *L. infantum* predicted proteome. Moreover, six proteins of *L. infantum* were selected which have predicted epitopes with affinity to 19 MHC alleles (human and mouse) of class I and affinity to at least 14 MHC (human and mouse) class II alleles. In addition, these proteins have also predicted B cell epitopes and were predicted to be secreted/excreted or plasma membrane proteins. Finally, for the selection of the peptides, a specific search was made in the relational database. We focused on the identification of specific epitopes of MHC molecules. Regarding MHC class I, the search criteria were restricted to identify binding epitopes, simultaneously, to the three most common human alleles of MHC class I (HLA-A2, HLA-B7 and HLA-B8), and mice alleles of MHC class I (H2-Db and H2-Dd). Human MHC class II (HLA-DRB1*0101, HLA-DRB1*0301, and HLA-DRB1*1501) and mice alleles (H2-IAb and H2-Iad) were prioritized to perform the bioinformatics analyses.

(2) Screening of the synthetic peptide using *L. infantum* naturally infected dogs: In vitro and in vivo screenings were performed to evaluate the capacity of these peptides to induce cellular proliferation, cytokine production by T-lymphocytes and a delayed-type hypersensitivity response in dog’s skin.

(3) Design of cocktail vaccines based on the peptides: After the screening in dogs, two peptide-based vaccines were designed (four peptides each) in association with a saponin adjuvant. Cockt-1 was designed based on the peptides with higher performance and Cockt-2 was designed using peptides with lower performance in vivo.

(4) Validation of peptide-based vaccine efficacy in the mouse model: The peptide-based vaccines were tested for immunogenicity, induction of polyfunctional T-cells, induction of memory T-cells and protective effects in mice.

### 2.3. L. infantum Naturally Infected Dog’s Selection for Peptide Screening

Five mongrel adult dogs, female and male, naturally infected with *L. infantum*, were used. They were kindly provided by the owners after signing the informed consent at the time of the animal retrieval by the Center for Zoonosis Control of Governador Valadares, Minas Gerais, Brazil, an endemic area for VL. The dogs were maintained in enclosed kennels, with access to water and balanced canine feed ad libitum, belonging to the Kennel of leishmaniasis in Universidade Federal de Ouro Preto, Minas Gerais, Brazil. They had direct contact with pen mates and received daily exercise and environmental enrichment in the facility. Furthermore, the animals were subjected to a quarantine protocol with broad-spectrum anthelmintic and were immunized against rabies (Tecpar, Curitiba, PR, Brazil) before the initiation of the screening. For selection of the dogs, we adopted the following criteria: (i) serological positive dogs by ELISA and DPP^®^ tests (recommended by the Brazilian government) and positive parasite isolation in NNN/LIT culture from bone marrow; (ii) dogs with a capacity to generate a cellular response in vivo through the Leishmanin skin test (LST) with formation of erythema and induration after phenol-treated *L. infantum* promastigotes stimulus; (iii) animals with a peripheral blood profile (leukogram) at the normality, following the clinical laboratory criteria recommended by Reis, et al. [8]; (iv) asymptomatic animals according to classification of Reis, et al. [9].

### 2.4. Dogs’ Sample Collection

Peripheral blood and skin samples were collected from the dogs. Regarding reverse antigen screening, skin biopsies were performed with aid of the tranquilizer acepromazine (1.1 mg/kg) and local blockage with 1% xylocaine using a 5 mm “punch”. The skin samples were obtained from the site of antigens injection and stored at −80 °C in Dimethyl sulfoxide/methanol until processing.

### 2.5. Parasites

Promastigotes of *L. infantum* strain OP46 (MCAN/*BR*/2008/*OP46*) maintained by passage in Syrian golden hamsters were cultured at 22–24 °C in medium LIT (liver infusion tryptose) with 100 U of penicillin G sodium and 100 μg of streptomycin sulfate per milliliter and sub-cultured in the same medium at an average density of 1 × 10^8^ cells/mL as described by Moreira, et al. [10]. The parasites were used for soluble *Leishmania* antigen (SLA) preparation as described by Reis, et al. [11], for LST preparation as described by Cardoso et al. [12] and for mice challenges.

### 2.6. Peptides

Thirty-eight *L. infantum* synthetic peptides (9–15 mer) were synthesized (Genscript, Piscataway, NJ, USA). Linear peptides were purified through a high-performance liquid chromatography approach with purity greater than 98%. All the synthetic peptides were resuspended in DMSO and stored at −80 °C until use. For the in vitro screening, peptides were randomized and combined into pools (with three peptides per pool). Pools were designed so that each peptide was repeated in two different pools. The 38 peptides were divided into 24 pools (A–X) as illustrated in Table S1. Due to the quantity of peptides to be screened we decided to use a combination matrix (as described by [13]) combining peptides into pools. To avoid the issue of a too large number of peptides in each pool we used a combination of three peptides per pool, where each peptide was repeated in two pools (Table S2). This strategy allowed us to reduce the number of antigens to be used, and also reduced the amount of biological samples of the dogs. Based on this combination matrix we were able to select the individual peptides.

### 2.7. In Vitro Peptide Screening—Lymphoproliferation and Intracellular IFN-γ Production

Dogs’ Peripheral blood mononuclear cells (PBMCs) were isolated from heparinized blood by density centrifugation (Histopaque-1077 and 1119; Sigma-Aldrich, St. Louis, MO, USA). The PBMCs (5 × 10^5^ cells/well) were CFDA-SE (Carboxyfluorescein diacetate succinimidyl ester)-labeled according to the Roatt, et al. [14] protocol and cultured in triplicate in a 48-well flat-bottom plate (Costar, Cambridge, MA, USA). The cells were stimulated or not with the synthetic peptide (1 μg/mL), SLA (100 μg/mL), and concavalin A (3 μg/mL) mitogen for 120 h at 37 °C and 5% CO_2_. After incubation, cells were treated with 10 μg/mL of brefeldin A (Sigma-Aldrich, St. Louis, MO, USA) for 4 h, then cells were removed, washed twice with Phosphate buffer saline (PBS), and stained at room temperature for 30 min in the dark with anti-CD4 APC (clone YKIX302.9) and anti-CD8 AF647 (clone YCATE55.9), all purchased from Biorad, Hercules, CA, USA. Afterwards, the cells were fixed with FACS fixing solution (10 g/L paraformaldehyde, 10.2 g/L sodium cacodylate, and 6.63 g/L sodium chloride, pH 7.2), then washed and treated with PBS buffer containing 0.5% saponin (Sigma) for permeabilization. After incubation with 10 μg/mL brefeldin A (Sigma-Aldrich, St. Louis, MO, USA) for 4 h at 37 °C, 5% CO_2_, cells were stained with anti-IFN-γ (clone CC302) and 50,000 events were acquired on the flow cytometer (FACScalibur, Becton Dickinson, San Jose, CA, USA). For the analyses FlowJo software (Becton Dickinson, San Jose, CA, USA) was used.

### 2.8. In Vivo Peptide Screening—Reverse Antigen Screening (RAS)

The parasite for LST was prepared according to Cardoso et al. [12]. Briefly, *L. infantum* promastigotes at an average density of 1 × 10^8^ cells/mL were phenol-treated. Then, phenol-treated promastigotes (10^8^), peptides (50 μg), and saline were intradermal administered with calibrated 1 mL syringes with 29.5 gauge swedged-on needles in the ventral region. Forty-eight hours later, dogs were sedated, and experienced readers used a ball point ink pen to place four cross marks around the reaction to accurately measure the length and width of induration and erythema (in mm) at each antigen site. Subsequently, considering that epitope conservancy plays a paramount role in the vaccine success, the conservancy of the eight selected epitopes was evaluated through an online available epitope conservancy tool* in the The immune epitope database (IEDB) analysis website [15].

### 2.9. Morphometrical and mRNA Expression Analyses of Dogs’ Skin Biopsies

Histological preparation and morphometrical analyses were performed as described by Cardoso, et al. [16]. The morphometrical analysis comprised the evaluation of inflammatory infiltrate by quantification of cellular nuclei by staining with hematoxylin and eosin.

Regarding mRNA expression analysis, specific primers for the follow cytokines were used: GAPDH (Forward-TTCCACGGCACAGTCAAG and Reverse-ACTCAGCACCAGCATCAC), IFN-γ (Fw-TCAACCCCTTCTCGCCACT and Rv-GCTGCCTACTTGGTCCCTGA), TNF-α (Fw-CGTCCATTCTTGCCCAAAC and Rv-AGCCCTGAGCCCTTAATTC), IL-4 (Fw-CACCTCCCAACTGATTCCAA and Rv-CTCGCTGTGAGGATGTTCAA), IL-10 (Fw-AGAACCACGACCCAGACATC and Rv-CCACCGCCTTGCTCTTATTC). RNA extraction, cDNA synthesis, and quantitative PCR (Polymerase chain reaction) to evaluate genes expression were carried out according to Roatt, et al. [17]. The analyses were performed in triplicate and normalized through constitutive gene glyceraldehyde-3-phosphate dehydrogenase (GAPDH). Results were expressed by the method 2^−∆∆Ct^.

### 2.10. Immunization Regimens and Vaccine Efficacy in the Mice Model

Regarding the vaccine efficacy, eight 6- to 8-week-old female BALB/c mice per group (Centro de Ciência animal (CCA) facility) were randomized by corporal weight and immunized three times biweekly subcutaneously in the back with 100 μL of vaccine formulations per mouse. The experimental groups were divided in SAL (animals that received sterile saline, 0.9% NaCl, pH 7.2–7.4), SAP (animals inoculated with 60 μg of saponin), Cockt-1 (animals that received 12.5 μg of each peptide: PEP17, PEP30, PEP33, and PEP34 associated with 60 μg of saponin) and Cockt-2 (animals inoculated with 12.5 μg of each peptide: PEP4, PEP12, PEP13, and PEP15 associated with 60 μg of saponin). Mice were challenged injecting 1.0 × 10^7^ *L. infantum* strain OP46 promastigotes intravenously 15 days after the last immunization. Parasite burden in the liver and spleen was analyzed 30 days after infection by real-time PCR (qPCR) quantification as described by Reis, et al. [18].

### 2.11. Polyfunctional T-Cell Analyses

Polyfunctional T cell phenotypes were assessed in splenocytes of mice 10 days after last immunization. Cell suspensions were incubated in RPMI supplemented with L-glutamine and fetal bovine serum and plated in 96-well round-bottom (Costar, Cambridge, MA, USA) culture plates at a concentration of 5 × 10^5^ cells/well. Cells were cultured for 24 hours at 37 °C with 5% CO_2_ in the presence of SLA (50 μg/mL). Brefeldin A (SIGMA) was added (10 μg/mL) at 20 hours of incubation. Afterwards, cells were blocked with anti-mouse CD16/CD32 (Mouse BD FC block^TM^, 0.5 μg/well) harvested, washed, treated with PBS plus an inert protein and stained with anti-mouse CD3 BV650 (clone 145.2C11), anti-mouse CD4 BV605 (clone RM4–5), anti-mouse CD8α BV786 (clone 53–6.7), and anti-mouse CD44 APC (clone IM7) (BD Biosciences Bioscience, USA) at room temperature for 30 min. The cells were fixed with FACS fixing solution, then washed and treated with PBS buffer containing 0.5% saponin for permeabilization. The cells were stained with anti-mouse IFN-γ FITC (clone XMG1.2, Bio-Rad), anti-mouse TNF-α PE-Cy7 (clone LG.3A10), and anti-mouse IL-2 PE (clone JES6-5H4) (Becton Dickinson, San Jose, CA, USA). The cells were acquired (300,000 events) on an LSR Fortessa (Becton Dickinson, San Jose, CA, USA) using FACSDiva software (Becton Dickinson, San Jose, CA, USA). For analysis in FlowJo software (Becton Dickinson, San Jose, CA, USA), dead cells were excluded after FVS510 staining and alive cells were gated for CD4^+^ and CD8^+^ T-cells and intracellular cytokine production. Polyfunctional cells were analyzed through Boolean gate strategy.

### 2.12. Proliferation Assay and Intracellular Cytokine Stain

The proliferation of antigen experienced T-cells was assessed by CFDA-SE assay 30 days after the mice challenge. Spleens harvested from the groups of mice and cell suspensions were prepared. Cell suspensions were incubated in 5 μM CFDA-SE for 10 min in RPMI without fetal bovine serum, followed by a block for 5 min in ice cold RPMI containing 10% Fetal bovine serum (FBS). CFDA-SE labeled cells were washed thoroughly before plating in a 96-well round-bottom (Costar, USA) culture plate at a concentration of 5 × 10^5^ cells/well. The cells were then cultured for 5 days at 37 °C with 5% CO2 in the presence of SLA (50 μg/mL). After incubation, the cells were treated with 10 μg/mL of brefeldin A (Sigma) for 4 h. Afterwards, the cells were blocked with anti-mouse CD16/CD32 (0.5 μg/well) and harvested, washed, treated with PBS plus an inert protein and stained with anti-mouse CD3 BV650 (clone 145.2C11), anti-mouse CD4 BV605 (clone RM4-5), and anti-mouse CD8α BV786 (clone 53–6.7) (BD Biosciences Bioscience, USA) at room temperature for 30 min. The cells were then fixed with FACS fixing solution, washed, and treated with PBS buffer containing 0.5% saponin for permeabilization. The cells were stained with anti-mouse IFN-γAF700 (clone XMG1.2), anti-mouse TNF-α PE-Cy7 (clone LG.3A10), and anti-mouse IL-2 PE (clone JES6-5H4) (BD Biosciences Bioscience, USA). The cells were acquired (300,000 events) on an LSR Fortessa (BD Biosciences, USA) using FACSDiva software. For analysis in FlowJo software, dead cells were excluded after FVS780 staining and alive cells were gated for CFSE stained CD4+ and CD8+ T-cells and intracellular cytokine production.

### 2.13. Analyses of T-Cell Memory Phenotypes through Flow Cytometry

Central and effector memory T-cells were analyzed 30 days post challenge. Splenocytes from animals were plated at 5 × 10^5^ cells/well in duplicate in 96-well round-bottom plates. The cells were then treated with the same conditions described above. After five days of culture, the cells were then prepared for flow cytometry analysis. The samples were blocked with anti-mouse CD16/CD32 (0.5 μg/well) and stained with surface markers at room temperature using the following antibodies: anti-mouse CD3 FITC (clone 17A2), anti-mouse CD4 BV605 (clone RM4-5), anti-mouse CD8α PerCP-Cy5.5 (clone 53–6.7), anti-mouse CD44 APC (clone IM7), anti-mouse CD45RA BV711 (clone 14.8), anti-mouse CD62L AF700 (clone MEL-14), anti-mouse CD127 BV510 (clone SB/199), and anti-mouse CD197 BV421 (clone 4B12). The cells were acquired (300,000 events) on an LSR Fortessa (BD Biosciences) using FACSDiva software. For analysis, dead cells were excluded after FVS780 staining and live cells were used.

### 2.14. Statistical Analysis

All data were analyzed through Graph Pad prism 7.0 software (Prism Software, Irvine, CA, USA) and expressed as mean plus standard deviation (SD). First, the normality of the data was assessed using the Shapiro–Wilk test and statistical differences were analyzed by one-way ANOVA and the Kruskal–Wallis test followed by Dunnett’s and Dunn’s multiple comparison tests, respectively. Correlation analyses were done by Pearson’s r test. Differences were considered significant when the *p*-value was <0.05.

## 3. Results

### 3.1. In Silico Analysis of L. infantum Predicted Proteome

Based on the published study concerning the mapping of potential *Leishmania* antigens through immunoinformatics [7], six *L. infantum* proteins from the predicted proteome were selected as candidate antigens. To avoid autoimmunity responses, peptides were checked for similarity with human, dog, and mouse proteomes. Thus, we selected thirty-eight peptides with high binding scores and no coverage identity to the above mentioned proteomes (Table S1).

### 3.2. Peptides Selected In Silico Trigger In Vitro Proliferation and IFN-γ Production by T-Lymphocytes in PBMC of L. infantum Naturally Infected Dogs

**Figure 1:**
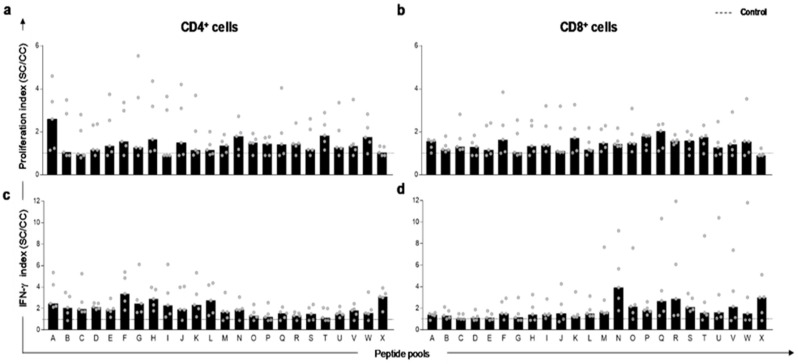
In vitro proliferation and IFN-γ production by T-lymphocytes in peripheral blood mononuclear cells (PBMC) of *L. infantum* naturally infected dogs after stimuli with predicted peptides. The spots represent indexes of T-cell responses to *Leishmania infantum* peptide pools in PBMC of naturally infected and asymptomatic dogs (*n* = 5 in gray circles). Proliferation index of CD4^+^ (**a**) and CD8^+^ (**b**) lymphocytes after peptide pool stimuli. (**c**) Index of IFN-γ producing by CD4^+^ and CD8^+^ (**d**) lymphocytes after peptide pool stimuli. Indexes were calculated based on SLA (soluble *Leishmania* antigen)-stimulated (SC) cultures divided by the control culture (CC). Cut-off (dashed line) for responses was calculated by the control culture indexes (cut-off = 1).

To evaluate the capacity of thirty-eight in silico-selected peptides to activate dog’s PBMC in natural infection, in vitro lymphoproliferation and IFN-γ production by specific PBMC T-cells were measured. The twenty-four peptide pools promoted specific CD4^+^ and CD8^+^ T-cell proliferation in dog’s PBMC after stimulus with *L. infantum* soluble antigen (Figure 1a,b). A higher rate of proliferation (2–6-fold in comparison to the control culture) was observed. Regarding IFN-γ production, peptide pools promoted an augment of this cytokine produced by CD4^+^ and CD8^+^ T-lymphocytes (Figure 1c,d), and this was observed by the indexes concerning unstimulated control culture. Thus, to select the individual peptide we used a combination matrix developed by us (Table S3). This matrix was based on scores for each pool which received 0, 1, 2, or 3 according to the indexes. Briefly, the value 0 was assigned for indexes less than 1.1, value 1 for indexes between 1.1 and 2, the value 2 for indexes between 2.1 and 3, and finally, the value 3 was assigned for indexes higher than 3.1. The criterion for pool selection was based on cut-off value. This value was determined according to the four evaluations performed in five dogs (the proliferation of CD4^+^ T-lymphocytes, proliferation of CD8^+^ T lymphocytes, production of IFN-γ by CD4^+^ T lymphocytes, and production of IFN-γ by CD8^+^ T-lymphocytes) in which we chose the minimum sum of 20. Therefore, to select a pool with satisfactory cut-off value, it would be necessary to obtain index values equal to or higher than 1 for each dog, which would represent indexes greater than 1.1 in all five dogs for the four parameters evaluated. This strategy allowed the selection of pools with better performance of lymphoproliferation and IFN-γ production in PBMC. Afterwards, the 13 best ranked pools of peptides were combined to select the ten finest peptides with immunogenic potential (PEP4, PEP12, PEP13, PEP15, PEP17, PEP25, PEP30, PEP33, PEP34, and PEP43).

### 3.3. Peptides Selected In Silico Lead to a Cellular Response in Skin of L. infantum Naturally Infected Dogs

**Figure 2:**
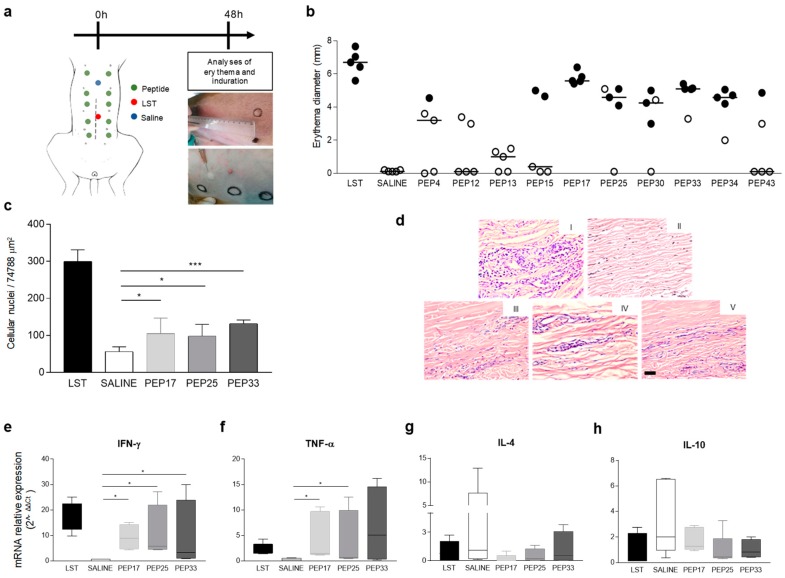
Cellular immune responses in dogs after inoculation with synthetic peptides. Naturally infected asymptomatic dogs (*n* = 5) were inoculated with 50 μg of synthetic peptides, and erythema and induration were measured 48 hours after injection. (**a**) Schematic and representative design of the reverse antigen screening approach by intradermal injection of saline, Leishmanin skin test (LST) and peptides in naturally infected dogs. (**b**) Erythema diameter with absence (o) and presence (●) of induration 48 h after injections. (**c**) Morphometric analysis of the inflammatory process for LST, saline, peptide 17 (PEP17), peptide 25 (PEP25), and peptide 33 (PEP33). (**d**) Representative Hematoxylin and eosin stains of biopsies taken from inoculums sites 48 h after LST (I), saline (II), PEP17 (III), PEP25 (IV), and PEP33 (V), black bar = 50 μm. (**e**–**h**) Box plots representing reverse-transcriptase quantitative PCR of mRNA expression for IFN-γ (**e**), TNF-α (**f**), IL-4 (**g**) and IL-10 (**h**) at the inoculum sites of LST, saline, PEP17, PEP25, and PEP33 injections. LST was not used for statistical analyses. The *p* values represent the difference between the groups saline, PEP17, PEP25, and PEP33: * *p-*value *<* 0.05, *** *p-*value *<* 0.0005.

Reverse antigen screening (RAS) was performed to assess the cellular response triggered by the peptides. Ten peptides previously selected in vitro were inoculated intradermally in dog’s abdomen as shown in Figure 2a. After 48 hours, the erythema diameter (with or without induration) formed at the inoculum site was measured. The results obtained after RAS are shown in Figure 2b. Negative control (saline) did not generate reaction at the inoculum site. In contrast, the positive control (Leishmanin skin test (LST)) generated strong reaction, as could be observed by the formation of erythema with induration (average diameter of 6.7 mm). Regarding the responses generated by the peptides, PEP17 showed a strong intradermal reaction, like LST, with erythema and induration (average diameter of 5.58 mm). PEP25, PEP30, PEP33, and PEP34 demonstrated important erythema formation with skin induration, although PEP25 and PEP30 showed no reaction in one dog. PEP4, although presenting a minor reaction, induced induration in one of the dogs. The other peptides did not present satisfactory results. Thus, peptides were ranked according to their ability to promote in vivo cellular immune response and subsequently we selected eight peptides that demonstrated higher and lower performance to compose two peptide-based vaccines. For subsequent analyses, we focused on PEP17, PEP25, and PEP33, comparing with the control groups (saline and LST). First, cellular infiltration in dog’s skin biopsies was evaluated through the morphometric approach. The analysis demonstrated that the positive control (LST), PEP17, PEP25, and PEP33 showed a significant increase (*p-*value < 0.05) in the quantitative evaluation of cell recruitment compared to the saline control (Figure 2c,d), although the peptides showed discrete inflammatory processes when compared to LST (*p-*value < 0.05). Afterwards, the mRNA expression levels of Th1, Th2, and IL-10 (immunoregulatory) cytokines were quantified (Figure 2e–h). We observed that PEP17, PEP25, PEP35, and LST induced significant mRNA expression of IFN-γ when compared with the saline group (Figure 2e). Similar results were found regarding TNF-α mRNA expression. In this regard, we observed that PEP17, PEP25, and LST promoted a significant enhancement in relation to the saline group as shown in Figure 2f. In contrast, concerning Th2 cytokine IL-4 and immunomodulatory IL-10, we did not observe differences between the saline group and the stimuli with LST, PEP17, PEP25, and PEP33 (Figure 2g,h).

After that, we performed the analyses of epitope conservancy and we observed that the peptides have a moderate conservancy across various *Leishmania* species (Table 1). PEP17 revealed 100% of sequence identity with *L. donovani*, *L. major*, *L. braziliensis*, and *L. amazonensis*. The other peptides revealed distinct sequence identity (66.67 to 100%) across the *Leishmania* species.

### 3.4. Peptide Cocktail Vaccine—Cockt-1 Enhances Frequency of Polyfunctional T-Cells in the Spleen of Immunized Mice

**Figure 3:**
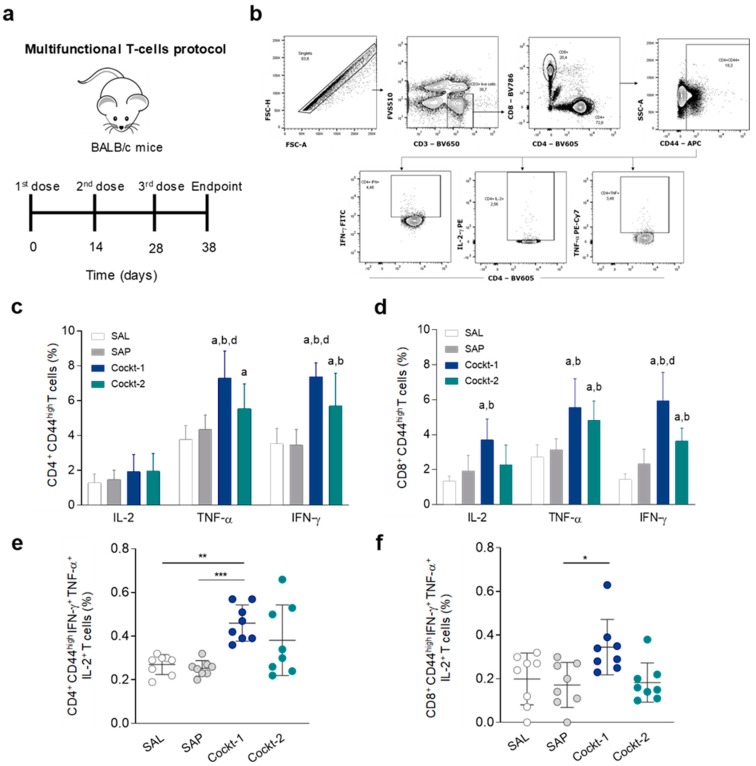
Frequency of polyfunctional T-cells in the spleens of immunized mice with peptide cocktail vaccine. Multifunctional T-cells were evaluated by frequency of intracellular IFN-γ, TNF-α, and IL-2-producing T-cells (activated T-cells that are CD44^high^) at the same time. (**a**) BALB/c mice were immunized three times and multifunctional T-cells were assessed in spleens ten days after last immunization using multicolor flow cytometry. (**b**) Representative plot of the gating strategy to characterize the multifunctional T-cells producer of intracellular IFN-γ, TNF-α, and IL-2 using Boolean gate strategy. (**c**) The frequency of CD4^+^CD44^high^ and (**d**) CD8^+^CD44^high^ cells producing IFN-γ, TNF-α, and IL-2 individually after in vitro stimulation with SLA (soluble *Leishmania* antigen). (**e**) The frequency of CD4^+^CD44^high^ IFN-γ^+^ TNF-α^+^ IL-2^+^ multifunctional cells after in vitro stimulation with SLA. (**f**) Frequency of CD8^+^CD44^high^ IFN-γ^+^ TNF-α^+^IL-2^+^ multifunctional cells after in vitro stimulation with SLA. Data are expressed as means ± SD of two independent experiments (*n* = 8). Significant differences between the groups are represented by the letters “a”, “b”, “c”, “d”, referring to groups SAL, SAP, Cockt-1, and Cockt-2, respectively; *p*-values: * *p-*value *<* 0.05, ** *p-*value *<* 0.005, *** *p-*value *<* 0.0005.

To determine if the cocktails of peptide-based vaccines enhance polyfunctional cytokine-producing by T-cells, we immunized mice three times (two weeks of interval) and ten days after the last dose the animals were sacrificed (Figure 3a). Using multicolor flow cytometry, an important population of cytokine-producing T-cells can be identified at the single-cell level, based on the combination of intracellular IL-2, IFN-γ, and TNF-α (Figure 3b). The analysis of single-cell cytokine production indicated that IFN-γ and TNF-α produced by CD4^+^ T-cells (Figure 3c) that were activated (CD44 ^high^ marker) are significantly higher in the Cockt-1 group when compared to the other groups (SAL, SAP, and Cockt-2). Also, it was observed that the Cockt-2 group displayed a higher level of TNF-α producing CD4^+^ T-cells when compared to the SAL group, and displayed a higher percentage of IFN-γ production than the SAL and SAP groups. Regarding cytokine production by CD8^+^ CD44^high^ T-cells (Figure 3d), IL-2 and TNF-α production by activated cells was shown as increased in the Cockt-1 group when compared to the control groups (SAL and SAP), whereas the IFN-γ production was higher than the other groups. The Cockt-2 group showed a high percentage of CD8^+^ CD44^high^ T-cell producing TNF-α and IFN-γ when compared to the control groups (SAL and SAP). Based on the importance of these cytokines in mediating protection, we carried out a comprehensive polyfunctional analysis of cytokine-producing T-cells. Using Boolean-gating strategy to combine the activation of CD4^+^ T cells (CD44^high^) and the triple cytokine production that is correlated to protection (Figure 3e), only Cockt-1 exhibited significant enhancement in the percentage of those cells when compared to the SAL and SAP groups. Regarding activated and multi-cytokine-producing CD8^+^ T-cells we observed a significant enhancement in Cockt-1 compared to the SAP group (Figure 3f).

### 3.5. Peptide Cocktail Vaccine Cockt-1 Promoted Significant T-Cell Proliferation in Spleen of Immunized and Challenged Mice

**Figure 4:**
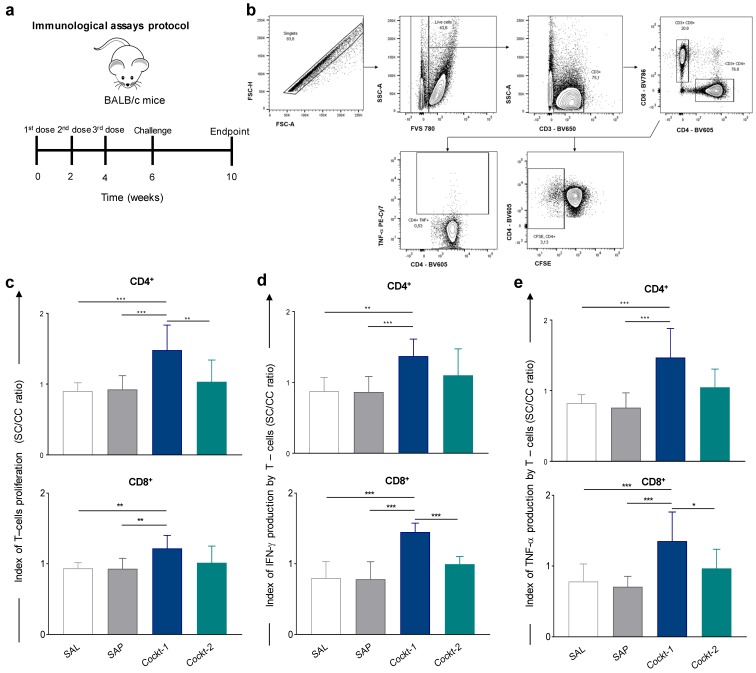
Spleen response after mice immunization with a cocktail of peptide-based vaccine and challenged with *L. infantum*. Cockt-1 promoted significant T-cell proliferation in spleens of immunized and challenged mice. (**a**) BALB/c mice were immunized three times and challenged with *L. infantum* promastigotes, four weeks after, splenocytes were obtained and T-cell proliferation and Th-1 cytokines were assessed through flow cytometry. (**b**) Representative plot of gating strategy to evaluate proliferation of T-cell subpopulations and IFN-γ and TNF-α–producing T-cells. (**c**) Plots represent index of T-cells proliferation (SC/CC ratio) of T-CD4^+^ and T-CD8^+^ cells after in vitro stimulation with SLA (soluble *Leishmania* antigen). (**d**) Plots represent the IFN-γ production indexes (SC/CC ratio) of T-CD4^+^ and T-CD8^+^ cells. (**e**) Plots represent the TNF-α production indexes (SC/CC ratio) of T-CD4^+^ and T-CD8^+^ cells after in vitro stimulation with SLA. Indexes were calculated based on SLA-stimulated (SC) cultures divided by the control culture (CC). Data are expressed as means ± SD of two independent experiments (*n* = 8). The *p*-values represent the difference between the groups: * *p-*value *<* 0.05, ** *p-*value *<* 0.005, *** *p-*value *<* 0.0005.

The efficiency of the synthetic peptides to induce specific immune responses in vivo was analyzed. For this, BALB/c mice were immunized with three doses of two peptide cocktails (two-week intervals) and then challenged. Thirty days after the challenge they were sacrificed (Figure 4a). The analysis strategies are shown in Figure 4b and results of intracytoplasmic cytokine production and proliferation of splenocyte lymphocytes were expressed as the culture index stimulated by the control culture (SC/CC). According to the results (Figure 4c), Cockt-1 induced a significant CD4^+^ T-cell proliferation when compared to the other groups (SAL, SAP, and Cockt-2) and higher CD8^+^ T-cell proliferation compared to the SAL and SAP groups (*p-*value < 0.05). Regarding IFN-γ production by CD4^+^ and CD8^+^ T-lymphocytes, we observed a significant increase (*p-*value < 0.05) in the Cockt-1 compared to the SAL, SAP, and Cockt-2 group (Figure 4d). In the TNF-α producing CD4^+^ and CD8 + T-cells analyses (Figure 4e), a significant increase in the Cockt-1 group can be observed when compared to the other groups analyzed.

### 3.6. The Cocktail 1 of Peptide-Based Vaccine Promoted a Reduction in Parasite Load in the Spleen of Vaccinated Mice and this Correlates with the Development of Central Memory and Effector Memory T-Cells

**Figure 5:**
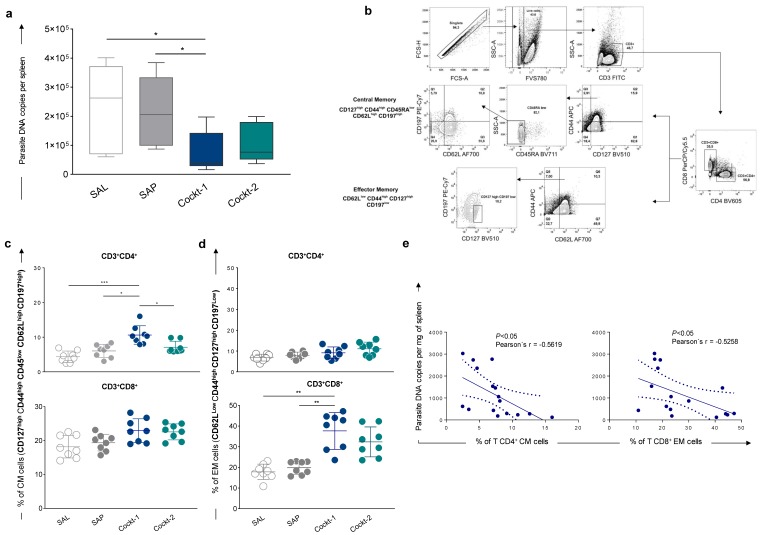
Parasite burden and memory correlation in spleens of immunized mice with a cocktail of peptide-based vaccine challenged with *Leishmania infantum*. BALB/c mice were immunized three times and challenged with *L. infantum* promastigotes, and then four weeks after, they were sacrificed for further analyses. (**a**) Parasite burdens were assessed in the spleen through quantitative PCR and results are expressed in parasite DNA copies per spleen. (**b**) Representative plot of gating strategy to evaluate central memory (CM) and effector memory (EM) T-cells. CM cells were characterized by CD3^+^ CD4^+^ or CD8^+^ CD127^high^ CD44^high^ CD45RA^low^ CD62L^high^ C197^high^ markers, and EM cells were characterized by CD3^+^ CD4^+^ or CD8^+^ CD62L^low^ CD44^high^ CD127^high^ C197^low^ after in vitro stimulation with SLA (soluble *Leishmania* antigen). Plots represent the frequency of CM T-cells (**c**) and EM T-cells (**d**) in mice splenocytes after in vitro stimulation with SLA. (**e**) Correlation analyses between parasite load and frequency of T CD4^+^ CM cells and T CD8^+^ EM cells are showed by Pearson’s r as means ± SD of two independent experiments (*n* = 8), and *p*-values represent the difference between the groups: * *p-*value *<* 0.05, ** *p-*value *<* 0.005, *** *p-*value *<* 0.0005.

To determine the level of cocktail peptide-based vaccine protection, BALB/c mice were immunized with three doses of two peptide cocktails (two-week intervals) and then challenged, and then thirty days after challenge they were sacrificed. Total spleen DNA was used for quantification of amastigotes copies using qPCR. The results are shown in Figure 5a, a significant reduction (*p-*value < 0.05) in parasite load in the Cockt-1 group was observed compared to the SAL and SAP groups. This reduction was approximately 70% for Cockt-1 when compared to the SAL group.

After that, to characterize memory phenotypes of T-cells, we investigated central (CM) and effector memory (EM) cells in the splenocytes culture using an extensive panel of superficial markers. CM T-cell phenotypes were characterized by CD127^high^ CD44^high^ CD45^low^ CD62L^high^ CD197^high^ and EM cells were characterized by CD62L^low^ CD44^high^ CD127^high^ CD197^low^, gating strategies are shown in Figure 5b. Results showed that after the challenge, the frequency of CM CD4^+^ T-cells is higher in the Cockt-1 group when compared to other groups (Figure 5c). We found a high frequency of EM CD8^+^ T-cells in the group vaccinated with Cockt-1 when compared to the control groups (SAL and SAP) as shown in Figure 5d. Moreover, a correlation analysis between the parasitic load and the frequency of central and effector T-lymphocytes found in the spleen was performed. Figure 5e shows the correlation analysis between the Cockt-1 group and the SAL group. A negative correlation (Pearson’s r = −0.5619; *p-*value < 0.05) was observed between the parasite load and the frequency of central memory CD4^+^ T-lymphocytes. In addition, we found a negative correlation (Pearson’s r = −0.5258, *p-*value < 0.05) between the parasite load and the frequency of effector memory CD8^+^ T-lymphocytes. In summary, when there is an increase in CD4^+^ T-lymphocytes central memory and CD8^+^ T-lymphocytes effector memory cells we observe a decrease in the parasitic load in this tissue.

## 4. Discussion

It is believed that an ideal vaccine against *Leishmania spp*. should have epitopes that will be recognized by APCs and will be able to trigger a T- lymphocyte effector response and maintain a long-lasting immune memory, which would be critical for protection against the parasite [6,19,20]. Therefore, the high-throughput search for epitopes with this potential has become one of the greatest current challenges for the rational design of vaccines. Thus, reverse vaccinology started to be used for this purpose, and one of the strategies used is the search for immunogenic epitopes in whole *Leishmania* proteomes [21].

Epitope mapping in proteomes of parasites is much more complex than the mapping of epitopes in proteins already described as immunogenic [21,22]. In this way, the use of the integrative approach to immunoinformatics proposed by Brito, Guimaraes, Velloso, Correa-Oliveira, Ruiz, Reis and Resende [7] was important to achieve potential epitopes in this study. After employing the immunoinformatics methodologies, we identified MHC class I and class II ligand epitopes comprising mouse and human alleles, due to the absence of predictors comprising dog alleles. However, based on some studies, we can verify that there are high levels of identity between MHC alleles of dogs and those of humans and mice [23,24]. For example, some loci of the MHC region II of dogs have an identity with those of humans and mice around 85% and 79%, respectively [23].

Regarding the in vivo screening of the immunoinformatics-selected peptides in the canine model, we selected asymptomatic dogs naturally infected by *L. infantum* (natural reservoir). Several studies have shown that dogs and humans with asymptomatic clinical forms have better reactivity with intra-dermal reaction than symptomatic individuals [25,26,27]. Similarly, Reis, et al. [28] have already shown that asymptomatic dogs have a profile of circulating lymphocytes in the peripheral blood capable of conferring a degree of resistance to the disease. When we performed the in vitro screening, the purpose was to identify potential epitopes in a huge number of peptides. For this, we focused on the main biomarkers of protection, namely: lymphocyte proliferation after stimulation with *L. infantum* and IFN-γ production by subpopulations of T-lymphocytes (CD4^+^ and CD8^+^). Currently, some markers related to protection have been studied, such as proinflammatory cytokines, production of immunoglobulins of IgG1 and IgG2 subtypes, and responsive CD4^+^ and CD8^+^ T-cells. The biomarkers that we chose are essential to determine resistance or susceptibility to visceral leishmaniasis and they are widely demonstrated in the literature [14,29]. Our data support that some peptides were able to induce lymphoproliferation and the production of IFN-γ by T-cells in vitro. In this regard, in many studies reporting the use of murine model and PBMC from patients with leishmaniasis, the main biomarker analyzed was IFN-γ produced after stimuli with different peptides [3,4,30].

After the in vitro screening, the reverse antigen screening (RAS) was used, a robust methodology that allows the selection of promising targets for the development of vaccines intended to trigger a strong cellular response [31]. Our findings demonstrate that an immunoinformatic approach increases the chance of finding peptides molecules (epitopes) which can trigger a cellular response in a naturally and asymptomatic infected model. The fact that an antigen generates a cellular response classified as type IV or late hypersensitivity implies that this antigen may be able to generate immune memory and trigger a response characterized by important cytokines, such as IFN-γ and TNF-α [27,32,33]. When we analyzed the epitope conservancy of these peptides among the proteome of other *Leishmania* species, it was observed that peptide PEP17 demonstrated to be the most conserved epitope among various viscerotropic and dermatotropic *Leishmania* species. Immunogenic peptides that show a high degree of conservancy with other parasites may be related to a better protection when compared to those not conserved [34]. In this way, peptides 17, 30, 33, and 34 may be considered promising epitopes for the design of vaccines against VL.

To validate the immunoinformatics approach and screening in the dog model, we designed two cocktail peptide-based vaccines according to RAS outcome in the canine model. We designed a cocktail based on peptides with the best performance in RAS (Cockt-1) and a second cocktail based on peptides with the worst performance (Cockt-2). Our data suggest that Cockt-1 can trigger spleen polyfunctional IL-2, TNF-α, and IFN-γ producing T-cells that are activated after the third immunization. The appearance of these polyfunctional T-cells is related to protection against the *Leishmania* parasite [35,36,37,38]. Some studies have already demonstrated that polyfunctional T-cells that produce these cytokines are more effective when compared to individual production for parasite elimination and control of infection [6,39,40,41]. Indeed, we observed that animals immunized with Cockt-1 can reduce the parasite load more than those not vaccinated. Moreover, these mice splenocytes can mount an in vitro response against *Leishmania infantum* soluble antigen by inducing proliferation of CD4^+^ and CD8^+^ T-lymphocytes and TNF-α and IFN-γ production. Although some peptides can be less immunogenic alone, when combined to other peptides or even the use of an adjuvant, this weakness can be bypassed, and they can promote a strong cellular response with intense proliferation of lymphocytes and production of important cytokines such as IFN-γ [3,5,34,42].

Based on the immunological findings in the spleen of Cockt-1 immunized mice, we assessed the parasite load in the endpoint to evaluate the efficacy of the peptide cocktails vaccine strategy. Regarding peptide vaccines for leishmaniasis, few studies are reported, and they are in immunogenicity phase with no outcome for parasite load [3,4,5,34,43,44]. Our results demonstrate that Cockt-1 can promote a significant reduction of parasite load in the spleen, which was not observed for Cockt-2. This demonstrates that the choice of immunogenic epitopes is the key for a successful vaccine. These results corroborate to Martins, et al. [45] that observed a decrease in parasitic load in the splenic compartment in the group immunized with a synthetic peptide (P2, which was mapped by immunoinformatics) and challenged with *L. infantum*. In another study using phages expressing *L. infantum* peptides, it was possible to observe a decrease in parasite load in the spleen [46]. Thus, the phages B1 and D11 promoted a reduction in the spleen parasite load relative to the saline group of 62% and 48%, respectively. Our results demonstrated a very similar reduction of 68% in the parasite load in the same organ when compared to the saline group.

Central and effector memory cells are fundamental to parasite elimination and disease control [47,48]. Central memory T-cells have an important role in the immune response, being responsible for the renewal of memory cells of the immune system while effector memory cells migrate to the infection site and eliminate the pathogen [49]. Our results of parasite reduction may be explained by the generation of memory T-cells in the animals immunized with Cockt-1. Thus, we can emphasize that both memory profiles are fundamental and participate directly in the control of the splenic parasite load. This becomes even more evident when we perform some correlation analyses in relation to the Cockt-1 and SAL groups. Regarding these analyses, we observed that when there is an increase in the frequencies of these memory phenotypes, there is a decrease in the parasitic load. We demonstrated that the larger frequency of memory T-cells at the time of recalling in the Cockt-1 group allows the mice to mount robust in vitro recall responses more willingly. Indeed, generation of larger numbers of memory T-cells is associated with enhanced protection upon a second encounter with the antigen.

Therefore, we show that peptides selected by immunoinformatics had promising outcomes in vitro and in vivo in the canine model with the signaling of protection markers against *L. infantum*. Markers such as the development of a cellular response by RAS, IFN-γ production and in vitro proliferation of T-cells could be observed. Regarding the phase I study in mice, Cockt-1 generated polyfunctional T -cells and memory T-cells and showed efficacy in reducing the parasite load on the splenic compartment. Thus, this emphasizes the promising field of peptide utilization in the development of vaccines against VL. In the future, to enhance the effectiveness of Cockt-1, we may employ modern methodologies such as nanotechnology, cellular vaccination, or even the design of polyepitope chimeras associated with an adjuvant system. Thus, the combination of immunogenic peptides with these recent methodologies seems to be the future for the design of a safe and effective vaccine against VL.

## Figures and Tables

**Table 1:** Conservancy analyses among various *Leishmania* species of the eight epitopes selected to compose peptide-based vaccines, using the IEDB conservancy tool.

Peptide ID	Sequence	% of Conservancy in *Leishmania* Species			
		*L. donovani*	*L. major*	*L. braziliennsis*	*L. amazonensis*
PEP4	QMVYNQDEI	100	100	100	88.98
PEP12	RLCPRGHSL	100	100	88.98	88.98
PEP13	QSGHNSGCL	100	100	88.98	100
PEP15	FALKRLSSL	100	100	66.67	100
PEP17	SVIHNATVV	100	100	100	100
PEP25	GGHFFFYVPPSPILF	93	80	80	80
PEP30	KGTTYPTTPNGLPSV	100	100	86.76	86.76
PEP33	IRQGFESFPPTPKTS	100	100	86.76	100
PEP34	GFESFPPTPKTSMM	100	100	86.76	100

## Supplementary Materials

The following are available online at https://www.mdpi.com/2076-393X/7/4/162/s1, Table S1: List of in silico predicted epitopes, alleles from MHC class I and II ligands derived from proteins of *Leishmania infantum* predicted proteome, Table S2: Strategy to combine the peptide into mixtures (Mix). The peptide pools are represented by letters (A–X) and the peptides by PEP followed by number, Table S3: Strategy (matrix) developed to interpret the results of the immune response triggered in vitro by the in silico-peptides in the PBMC of five (C03, C16, C20, C25, and C29) naturally infected dogs. The peptide pools are represented by letters (A–X) and the selected pools are highlighted by black color (highest scores).
