# Supplementary material for: Synthetic Peptides Elicit Strong Cellular Immunity in Visceral Leishmaniasis Natural Reservoir and Contribute to Long-Lasting Polyfunctional T-Cells in BALB/c Mice
Source: Vaccines (Basel). 2019 Oct 28;7(4):162. doi: 10.3390/vaccines7040162 (PMC6963447; doi:10.3390/vaccines7040162)
Supplement: Supplementary file 1 [file vaccines-07-00162-s001.docx]

**Table S1.** List of *in silico* predicted epitopes, alleles from MHC class I and II ligands derived from proteins of *Leishmania infantum* predicted proteome**.**

| **Pep_ID** | **Sequence** | **Position** | **Binding** | **Allele** | **Protein** |
| --- | --- | --- | --- | --- | --- |
| PEP1 | LVVSIPIAL | 303 | MHC I | HLA-A2; HLA-B7 | LinJ.18.1500 [XP_001464963](https://www.ncbi.nlm.nih.gov/protein/XP_001464963) |
| PEP2 | YLLARFYET | 283 |  | HLA-A2; HLA-B8 |  |
| PEP3 | KPQRRQSVL | 29 |  | HLA-B7; HLA-B8; H2-Ld |  |
| PEP4 | Ac-QMVYNQDEI | 453 |  | H2-Db |  |
| PEP5 | RGYTCAMTG | 596 |  | H2-Dd |  |
| PEP25 | GGHFFFYVPPSPILF | 810 | MHC II | H2-IAb |  |
| PEP26 | FFFYVPPSPILFCGA | 813 |  | H2-IAb |  |
| PEP6 | YLRLWALSL | 798 | MHC I | HLA-A2; HLA-B7; HLA-B8; B32; HLA-B39 | LinJ.32.0970 [XP_001467777](https://www.ncbi.nlm.nih.gov/protein/XP_001467777) |
| PEP7 | FMLLGMPYI | 652 |  | H2-Db |  |
| PEP8 | SGYTWAPIS | 489 |  | H2-Dd |  |
| PEP27 | EYVLSSVSNTASYLR | 786 | MHC II | DRB1_0101; DRB1_0701; DRB1_1501; DRB1_1302; DRB1_0901; DRB1_0404; DRB1_0401 |  |
| PEP28 | TSFVVLCASPTMIVR | 235 |  | DRB1_0101; DRB1_0701; DRB1_1501; DRB1_1302; DRB1_0901 |  |
| PEP29 | QKGTTYPTTPNGLPS | 499 |  | H2-IAb |  |
| PEP30 | KGTTYPTTPNGLPSV | 500 |  | H2-IAb |  |
| PEP9 | LAYDHRAYL | 215 | MHC I | HLA-A2; HLA-B7; HLA-B8; HLA-B39; B58; H2-Kb | LinJ.36.2160 [XP_001469796](https://www.ncbi.nlm.nih.gov/protein/XP_001469796) |
| PEP10 | PARDNEPKL | 423 |  | H2-Db |  |
| PEP11 | TLPFWRVAW | 190 |  | H2-Db |  |
| PEP32 | TVATWVLLWRTRWAA | 262 | MHC II | DRB1_0101; DRB1_0701; DRB1_1501; DRB1_0405; DRB1_0802; DRB5_0101 |  |
| PEP33 | IRQGFESFPPTPKTS | 377 |  | H2-IAb |  |
| PEP34 | GFESFPPTPKTSMM | 37 |  | H2-IAb |  |
| PEP12 | RLCPRGHSL | 119 | MHC I | HLA-B7; HLA-B8; HLA-B62 | LinJ.36.5700 [XP_001469929](https://www.ncbi.nlm.nih.gov/protein/XP_001469929) |
| PEP13 | Ac-QSGHNSGCL | 97 |  | H2-Db |  |
| PEP14 | IGPGSALAL | 353 |  | H2-Dd |  |
| PEP35 | PRVTVPATMDKKMLL | 454 | MHC II | DRB1_0101; DRB1_0301 |  |
| PEP37 | VATAREIRQKYPRKR | 69 |  | DRB1_0101 |  |
| PEP15 | FALKRLSSL | 25 | MHC I | HLA-A2; HLA-B7; HLA-B8 | LinJ.28.1850 XP_001470212 |
| PEP16 | YWYWYPYLL | 791 |  | HLA-A2; HLA-HLA-A24; HLA-B39; HLA-B7 |  |
| PEP17 | SVIHNATVV | 379 |  | H2-Db |  |
| PEP18 | RGGSRMVAF | 247 |  | H2-Dd |  |
| PEP38 | FYVANVLLQDITFRR | 189 | MHC II | DRB1_0101; DRB1_0301; DRB1_1501; DRB1_0401; DRB1_0901; DRB1_1302 |  |
| PEP40 | GAPVAAAVPESPEQK | 324 |  | H2-IAb |  |
| PEP19 | YLQPSRTRL | 332 | MHC I | HLA-A2; HLA-B7; HLA-B8 | LinJ.19.0350 XP_001464998 |
| PEP20 | KMLTKSSIM | 294 |  | HLA-A2; HLA-B62; HLA-B8 |  |
| PEP21 | SAVTLGQYL | 325 |  | H2-Db |  |
| PEP22 | RPPPLDPEE | 173 |  | H2-Dd |  |
| PEP42 | EMGVDYIVMTMVDRD | 191 | MHC II | DRB1_0101; DRB1_1501; DRB1_0405; DRB1_1302; DRB5_0101; DRB1_0405; DRB1_1302; DRB5_0101 |  |
| PEP43 | LVEQLAVTPLSVYAH | 244 |  | H2-IAb |  |
| PEP44 | LKLVEQLAVTPLSVY | 242 |  | DRB1_0101; DRB1_1501; DRB1_0701; DRB1_1302; H2-IAb |  |

**Table S2.** Strategy to combine the peptide into mixtures (Mix). The peptide pools are represented by letters (A-X) and the peptides by PEP followed by number

| **Pool** | **D** | **E** | **F** | **Pool** | **P** | **Q** | **R** |
| --- | --- | --- | --- | --- | --- | --- | --- |
| **A** | PEP4 | PEP5 | PEP25 | **M** | PEP22 | PEP35 | PEP39 |
| **B** | PEP8 | PEP9 | PEP27 | **N** | PEP19 | PEP20 | PEP40 |
| **C** | PEP10 | PEP11 | PEP28 | **O** | PEP21 | PEP41 | PEP42 |
| **Pool** | **J** | **K** | **L** | **Pool** | **V** | **W** | **X** |
| **G** | PEP12 | PEP13 | PEP29 | **S** | PEP18 | PEP43 | PEP6 |
| **H** | PEP14 | PEP30 | PEP33 | **T** | PEP37 | PEP35 | PEP32 |
| **I** | PEP15 | PEP17 | PEP34 | **U** | PEP2 | PEP26 | PEP7 |

**Table S3** Strategy (matrix) developed to interpret the results of the immune response triggered *in vitro* by the *in silico*-peptides in the PBMC of five (C03, C16, C20, C25, and C29) naturally infected dogs. The peptide pools are represented by letters (A-X) and the selected pools are highlighted by black color (highest scores)

|  | **Index (CE/CC) of CD4^+^ proliferation** | | | | | **Index (CE/CC) of CD8^+^ proliferation** | | | | | **Index (CE/CC) of CD4^+^ producing IFN-γ** | | | | | **Index (CE/CC) of CD8^+^ producing IFN-γ** | | | | |  |
| --- | --- | --- | --- | --- | --- | --- | --- | --- | --- | --- | --- | --- | --- | --- | --- | --- | --- | --- | --- | --- | --- |
|  | C03 | C16 | C20 | C25 | C29 | C03 | C16 | C20 | C25 | C29 | C03 | C16 | C20 | C25 | C29 | C03 | C16 | C20 | C25 | C29 | Score |
| POOL A | 1 | 1 | 3 | 3 | 3 | 1 | 0 | 1 | 1 | 1 | 2 | 2 | 3 | 2 | 3 | 1 | 1 | 0 | 1 | 1 | 31 |
| POOL B | 0 | 0 | 3 | 0 | 2 | 1 | 1 | 0 | 1 | 1 | 2 | 3 | 3 | 0 | 1 | 1 | 1 | 1 | 2 | 0 | 23 |
| POOL C | 0 | 0 | 2 | 0 | 2 | 1 | 1 | 1 | 1 | 2 | 2 | 1 | 3 | 1 | 1 | 1 | 1 | 0 | 0 | 0 | 20 |
| POOL D | 1 | 1 | 2 | 0 | 2 | 0 | 1 | 0 | 1 | 1 | 1 | 2 | 3 | 2 | 2 | 0 | 1 | 1 | 0 | 1 | 22 |
| POOL E | 1 | 0 | 3 | 0 | 2 | 0 | 1 | 0 | 2 | 2 | 1 | 1 | 2 | 1 | 1 | 1 | 1 | 0 | 0 | 1 | 20 |
| POOL F | 0 | 1 | 3 | 1 | 2 | 0 | 0 | 1 | 2 | 3 | 3 | 1 | 3 | 3 | 2 | 1 | 2 | 0 | 1 | 2 | 31 |
| POOL G | 0 | 1 | 3 | 1 | 3 | 0 | 0 | 0 | 1 | 2 | 1 | 1 | 3 | 2 | 2 | 0 | 3 | 0 | 1 | 1 | 25 |
| POOL H | 1 | 1 | 3 | 0 | 3 | 0 | 0 | 1 | 2 | 2 | 2 | 2 | 3 | 3 | 1 | 0 | 3 | 0 | 1 | 2 | 30 |
| POOL I | 0 | 0 | 3 | 0 | 3 | 1 | 1 | 0 | 2 | 3 | 2 | 1 | 3 | 3 | 1 | 1 | 2 | 0 | 1 | 1 | 28 |
| POOL J | 0 | 1 | 3 | 0 | 3 | 0 | 0 | 0 | 2 | 3 | 1 | 1 | 3 | 3 | 0 | 0 | 3 | 0 | 2 | 1 | 26 |
| POOL K | 0 | 1 | 2 | 0 | 3 | 1 | 0 | 1 | 2 | 3 | 2 | 1 | 3 | 3 | 1 | 1 | 3 | 1 | 1 | 1 | 30 |
| POOL L | 0 | 1 | 3 | 0 | 3 | 1 | 0 | 2 | 1 | 1 | 2 | 1 | 3 | 3 | 0 | 1 | 3 | 1 | 1 | 1 | 28 |
| POOL M | 1 | 1 | 0 | 1 | 0 | 0 | 1 | 1 | 2 | 2 | 1 | 0 | 3 | 1 | 1 | 3 | 1 | 1 | 1 | 2 | 23 |
| POOL N | 2 | 1 | 0 | 1 | 1 | 1 | 1 | 1 | 2 | 1 | 1 | 0 | 1 | 1 | 3 | 3 | 2 | 3 | 1 | 3 | 29 |
| POOL O | 1 | 1 | 0 | 1 | 1 | 0 | 1 | 1 | 3 | 1 | 1 | 0 | 2 | 1 | 1 | 3 | 2 | 1 | 0 | 2 | 23 |
| POOL P | 1 | 1 | 0 | 0 | 1 | 1 | 1 | 1 | 1 | 1 | 1 | 0 | 2 | 1 | 0 | 3 | 2 | 1 | 1 | 1 | 20 |
| POOL Q | 1 | 0 | 0 | 1 | 3 | 1 | 1 | 2 | 2 | 2 | 1 | 0 | 2 | 1 | 1 | 3 | 1 | 3 | 1 | 2 | 28 |
| POOL R | 2 | 1 | 0 | 1 | 1 | 1 | 1 | 1 | 1 | 1 | 1 | 0 | 1 | 1 | 1 | 3 | 1 | 3 | 1 | 2 | 24 |
| POOL S | 2 | 1 | 2 | 0 | 1 | 1 | 0 | 2 | 1 | 1 | 2 | 0 | 2 | 0 | 1 | 3 | 1 | 3 | 1 | 2 | 26 |
| POOL T | 2 | 1 | 2 | 1 | 1 | 1 | 0 | 1 | 2 | 1 | 2 | 0 | 0 | 1 | 2 | 3 | 1 | 2 | 1 | 0 | 24 |
| POOL U | 3 | 0 | 1 | 2 | 1 | 1 | 0 | 0 | 2 | 1 | 2 | 1 | 1 | 1 | 1 | 3 | 1 | 3 | 1 | 0 | 25 |
| POOL V | 3 | 0 | 1 | 1 | 1 | 1 | 0 | 1 | 2 | 1 | 1 | 0 | 1 | 1 | 2 | 3 | 1 | 3 | 1 | 0 | 24 |
| POOL W | 2 | 0 | 1 | 2 | 1 | 1 | 0 | 0 | 3 | 1 | 3 | 0 | 1 | 1 | 1 | 3 | 0 | 3 | 1 | 0 | 24 |
| POOL X | 1 | 1 | 0 | 0 | 0 | 0 | 0 | 0 | 0 | 1 | 3 | 1 | 3 | 3 | 3 | 3 | 0 | 3 | 3 | 1 | 26 |
